# Exploring the Relationship Between 91 Inflammatory Cytokines and IgA Nephropathy Using a Two-Sample Mendelian Randomization Study and the Gene Expression Omnibus Database

**DOI:** 10.1155/mi/5142090

**Published:** 2025-04-26

**Authors:** Manyi Wu, Xingxin Yang, Mengxiao Zou, Xiaojing Cai, Chunyu Pan, Junhua Li, Shuwang Ge

**Affiliations:** Department of Nephrology, Tongji Hospital, Tongji Medical College, Huazhong University of Science and Technology, Wuhan, China

**Keywords:** IgA nephropathy, inflammatory cytokines, Mendelian randomization

## Abstract

**Objectives:** Previous research has demonstrated associations between various inflammatory cytokines and IgA nephropathy (IgAN). However, the causal relationships between them remain unclear. The purpose of this study is to extensively analyze the causal links between 91 circulating cytokines and IgAN.

**Methods:** This study commenced with a two-sample bidirectional Mendelian randomization analysis. Genetic variations associated with 91 circulating inflammatory cytokines were extracted from genome-wide association study (GWAS) data involving individuals of European ancestry (*n* = 14824). In the corresponding GWAS dataset, the genetic variations for IgAN were obtained from a Finnish cohort of European ancestry, consisting of a case group (*n* = 653) and a control group (*n* = 411528). The findings from the Mendelian randomization analysis were subsequently subjected to preliminary validation using the GSE116626 dataset from the GEO database.

**Results:** Our MR analysis indicates that transforming growth factor-alpha (TGF-alpha), leukemia inhibitory factor (LIF), and C–C motif chemokine 19 (CCL19) are linked to an increased risk of IgAN. There were no causal connections found when IgAN was used as an exposure and the 91 circulating inflammatory cytokines as outcomes. In addition, the GSE116626 dataset from the GEO database revealed significant upregulation of CCL19 in renal tissues from patients diagnosed with IgAN.

**Conclusions:** This study shows a causal link between inflammatory cytokines and IgAN, suggesting that TGF-alpha, LIF, and CCL19 may act as upstream mediators in the pathogenic pathways of IgAN. The critical role of CCL19 in the pathogenesis of IgAN was further validated using data from the GEO database. However, whether these cytokines can be used to predict or ameliorate the progression of IgAN requires further investigation.

## 1. Introduction

IgA nephropathy (IgAN) is the world's most prevalent primary glomerular disease and the primary cause of kidney failure. A large cohort study by British researchers found that even among IgAN patients with low progression risk and minimal proteinuria, 50% experienced renal failure or died during the follow-up period [1].

The “four-hit hypothesis” is a well-established model for the pathogenesis of IgAN. It posits that immune complexes, once formed, deposit within the renal mesangium, activating mesangial cells and initiating the local overproduction of cytokines, chemokines, and complement, which ultimately leads to podocyte injury and proteinuria, thereby intensifying chronic inflammation [2]. Inflammation is central to the pathophysiology of IgAN, with multiple cytokines mediating inflammatory damage [3–5]. Cytokines, tiny proteins with a variety of biological functions, are created and released by immune and non-immune cells in reactions to varied stimuli.

They perform functions ranging from immune regulation and cell growth to tissue repair [6] and are classified as interleukins, chemokines, and growth factors, among others. However, whether cytokines are causal factors or merely consequences of IgAN remains under debate. This ambiguity stems from potential biases in observational studies due to disease progression, post-diagnosis treatments, and confounding factors, including reverse causality. These complexities complicate the establishment of a definitive causal relationship.

Mendelian randomization (MR) leverages genetic variants strongly associated with exposure factors as instrumental variables (IVs) to infer causal relationships between exposures and outcomes [7]. When compared to observational research, MR analysis reduces the influence of reverse causality and confusion, offering stronger evidence for causal inference [8]. In this study, valid genetic variants were initially identified from summary data of 91 published genome-wide association studies (GWASs) on inflammatory cytokines to assess their associations with IgAN. Reverse exposure and outcome analyses were used to further investigate the causal relationship, providing evidence for targeting specific inflammatory cytokines in the treatment of IgAN.

The rapid development of bioinformatics and high-throughput sequencing technologies has led to the growing use of integrative bioinformatics approaches in nephrology research. In earlier studies, microarray expression profiling of peripheral blood samples from IgAN patients was conducted to identify hub genes associated with IgAN [9, 10]. Differentially expressed genes (DEGs) are defined as those exhibiting significantly different expression levels across various biological samples or conditions. The analysis of DEGs facilitates the exploration of disease mechanisms and the identification of potential biomarkers and therapeutic targets [11].

In this study, MR analysis was initially used to investigate the relationships between 91 inflammatory cytokines and IgAN, identifying three cytokines with causal relationships to IgAN. Subsequently, we intersected the MR findings with GEO datasets related to IgAN to explore the specific roles of these cytokines in the disease's pathogenesis. The analysis revealed one cytokine that was positively expressed in the Gene Expression Omnibus (GEO) database. This result not only confirms the pivotal role of this cytokine but also provides critical insights for further investigation into its underlying mechanisms and suggests a new potential target for IgAN diagnosis and treatment.

## 2. Methods

### 2.1. Ethical Approval and Participant Consent

The data used in this investigation came from two previously published GWAS studies as well as an international public genetic data repository. The ethical committees authorized both GWAS investigations, and all subjects provided informed written consent.

### 2.2. Mendelian Randomization Hypothesis

A bidirectional two-sample MR method was employed in this research. The MR design is founded on three key assumptions: (1) relevance: the genetic variant is strongly associated with the exposure; (2) independence: the genetic variant is not linked to confounding factors that impact both the exposure and the outcome; and (3) specificity: the genetic variant influences the outcome only through the exposure, without involving alternative mechanisms [12]. This research employed publicly available GWAS data on 91 circulating inflammatory cytokines and IgAN. First, genetic instruments for each circulating inflammatory cytokine were chosen to investigate its causal links with IgAN. Second, genetic instruments associated with IgAN were used to infer causal relationships between IgAN and each circulating inflammatory cytokine.

### 2.3. Genome-Wide Association Study Data of IgA Nephropathy

The data used for the IgAN analysis in this study were obtained from the FinnGen project, a large intersectoral partnership research program. The IgAN dataset includes 653 cases and 14824 European ancestry controls, diagnosed according to ICD-10 criteria.

### 2.4. Genome-Wide Association Study Data of 91 Circulating Inflammatory Cytokines

We used data from a study that found genome-wide relationships between genetic variants and 91 plasma proteins, which evaluated a cohort containing 14824 European ancestors. The GWAS summary data from this research were formerly released in the Journal of Nat Immunol [13] (GCST90274758-GCST90274848) (Supporting Information Table S1).

### 2.5. Selection of Genetic Instrumental Factor

Initially, we used a criterion of *p*  < 5 × 10^−8^ to screen single-nucleotide polymorphisms (SNPs) closely associated with circulating inflammatory cytokines and IgAN. However, when certain cytokines were treated as exposures, only a limited number of SNPs were identified. Therefore, a less stringent threshold of *p*  < 5 × 10^−6^ was adopted to identify genetic instruments for the exposures. In addition, linkage disequilibrium among these instruments was addressed to ensure their independence (*r*^2^ < 0.001, kb = 10,000). The *F*-statistic was calculated for each SNP to assess potential weak instrument bias, with *F* > 10 indicating no significant bias [14].

### 2.6. Statistical Analysis

This study employs five MR analysis methods, with inverse variance weighting (IVW) as the primary approach, supplemented by weighted median, MR-Egger, weighted mode, and simple mode analyses. The IVW method omits the intercept in the regression and fits the model using the inverse of the outcome variance (square of the standard error) as the weight, assuming that all SNPs are valid IVs with a total bias of zero [15]. MR-Egger [16] regression accounts for the intercept, based on the assumption that the instrument exposure and instrument outcome associations, are independent—meaning that instrument strength is unrelated to direct effects. The weighted median [17] approach requires that at least 50% of the instruments are valid for a robust estimate. If IVW analysis shows significant results (*p*  < 0.05) without horizontal pleiotropy or heterogeneity, the results are regarded as positive—even if other approaches do not provide significant outcomes—provided that the direction of the *β* coefficient remains consistent [18].

Before applying these methods, we carefully evaluated the core assumptions underlying each approach and the validity of our IVs. We specifically assessed the strength of the instruments by calculating the *F* statistic for each IV (all *F* > 10, Supporting Information Table S1), thereby fulfilling the basic requirements for both the IVW and MR-Egger methods. Furthermore, horizontal pleiotropy was systematically evaluated using the MR-Egger intercept test (*p*  > 0.05) and the MR-PRESSO global test, which identified no significant outlier SNPs (Table 1). These results collectively indicate that the selected instruments are robust and free from systematic bias.

To evaluate the MR results' robustness, we used three sensitivity analysis techniques. Heterogeneity was assessed using Cochran's Q test [19], with a *p*-value greater than 0.05 indicating the absence of heterogeneity. The MR-Egger [16] intercept test further evaluated horizontal pleiotropy, with a *p*-value above 0.05 supporting the absence of pleiotropy. In addition, the MR-PRESSO [20] method was applied for simultaneous outlier detection and pleiotropy testing. The leave-one-out sensitivity analysis examined whether any single SNP associated with both the exposure and outcome disproportionately influenced the causal relationship. All analyses were conducted using R version 4.3.3 along with the “TwoSampleMR” and “MR-PRESSO” packages.

### 2.7. Validation of Gene Expression Omnibus

The National Center for Biotechnology Information (NCBI) [21] developed and maintained the high-throughput gene expression database known as GEO since 2000. Using “IgA nephropathy” as the keyword, we screened and downloaded the dataset GSE116626 from the GEO database, which contained 52 kidney tissues from IgA nephropathy and 7 kidney tissues from the control group. DEGs for IgA nephropathy were obtained using the “limma” package in R software with |LogFC≥1.5|, *p*  < 0.05. The intersection of MR results and GEO DEGs was taken, and the intersection results were analyzed by GraphPad Prism software. Protein–protein interaction (PPI) network was constructed by analyzing the protein interactions of CCL19 through the STRING platform, and the condition of “interaction score ≥ 0.4” was used to construct the PPI. An unpaired *t*-test was used for statistical methods, and *p*  < 0.05 was considered statistically different.

## 3. Results

### 3.1. Genetic Predictors of 91 Circulating Inflammatory Cytokines and IgA Nephropathy

When screening SNPs linked to each circulating inflammatory cytokine and IgAN, a significance threshold of *p*  < 5 × 10^−6^ was chosen to guarantee a sufficient number of SNPs for ensuing MR analysis. All IVs used had *F*-statistics greater than 10, indicating the absence of weak instruments.

### 3.2. Investigating the Relationship Between 91 Circulating Inflammatory Cytokines and the IgA Nephropathy

**Figure 1:**
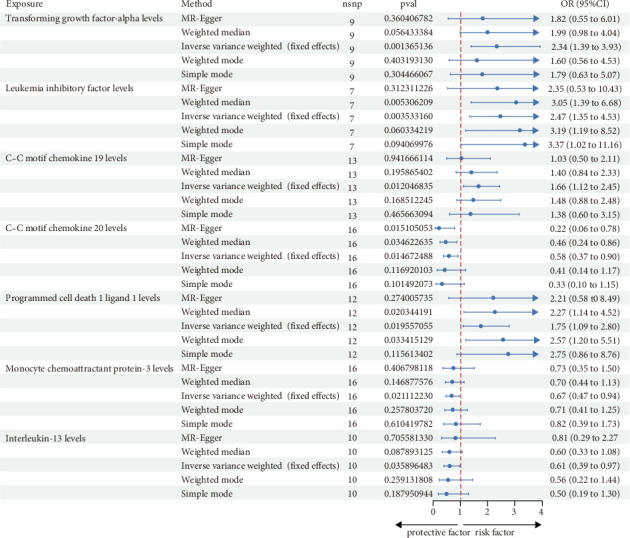
Forest plot showing the results of five methods of MR analysis of the causal relationship between 91 inflammatory cytokines and IgAN.

When considering the 91 circulating inflammatory cytokines as exposures and IgAN as the outcome, the analysis results are shown in Figure 1. Seven genetically predicted inflammatory cytokines were associated with IgAN, with transforming growth factor-alpha levels (TGF-alpha, OR: 2.34, 95% CI: 1.39–3.93, *p*=0.0013), leukemia inhibitory factor (LIF) levels (OR: 2.47, 95% CI: 1.35–4.53, *p*=0.0035), C–C motif chemokine 19 (CCL19) levels (OR: 1.66, 95% CI: 1.12–2.45, *p*=0.012), and programmed cell death 1 ligand 1 levels (OR: 1.75, 95% CI: 1.09–2.80, *p*=0.019) serving as promoters, while C–C motif chemokine 20 levels (OR: 0.58, 95% CI: 0.37–0.91, *p*=0.014), monocyte chemoattractant protein-3 levels (OR: 0.67, 95% CI: 0.47–0.94, *p*=0.021), and Interleukin-13 levels (OR: 0.61, 95% CI: 0.39–0.97, *p*=0.035) acted as inhibitors.

Among these, the MR-Egger analysis for LIF levels also showed a *p*-value < 0.05. C–C motif chemokine 20 levels did not pass the MR-PRESSO analysis, and programmed cell death 1 ligand 1 levels, interleukin-13 levels, and monocyte chemoattractant protein-3 levels did not pass the leave-one-out analysis (Table 1, Supporting Information Figure S1). In the MR-Egger analysis, no signs of horizontal pleiotropy were observed for TGF-alpha levels, LIF levels, and CCL19 levels, and all three passed the MR-PRESSO test.

**Figure 2:**
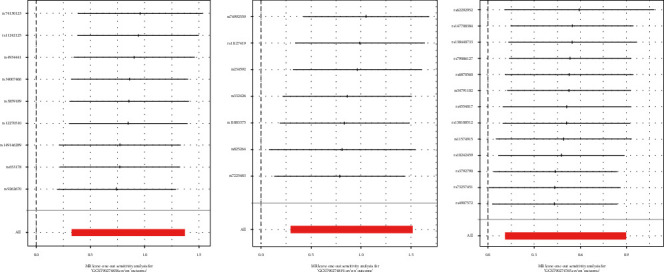
Leave-one-out plots of the causal relationship between TGF-alpha (GCST90274838), LIF (GCST90274819), and CCL19 (GCST90274765) and IgAN.

**Figure 3:**
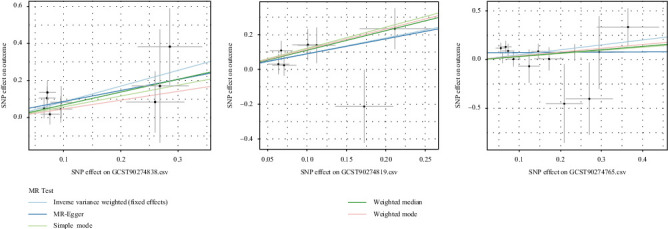
Scatterplot of TGF-alpha (GCST90274838), LIF (GCST90274819), and CCL19 (GCST90274765) versus IgAN causality.

The robustness of the findings was validated through the leave-one-out analysis (Figure 2), with consistent directions of the *β* coefficients across the MR-Egger, simple model, weighted median, and weighted mode methods.  Figure 3 presents the scatter plots illustrating their causal relationships with IgAN.

### 3.3. Investigating the Relationship Between IgA Nephropathy and 91 Circulating Inflammatory Cytokines

When IgAN was taken as the exposure and the 91 circulating inflammatory cytokines as the outcomes, no evidence of causal links was found.

### 3.4. Validation of Gene Expression Omnibus

**Figure 4:**
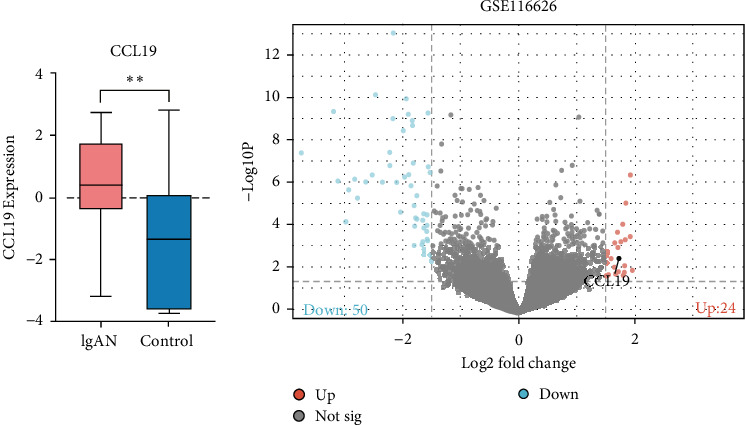
Comparison of CCL19 gene expression in IgAN nephropathy tissues (*⁣*^*∗∗*^*p＜*0.01).

**Figure 5:**
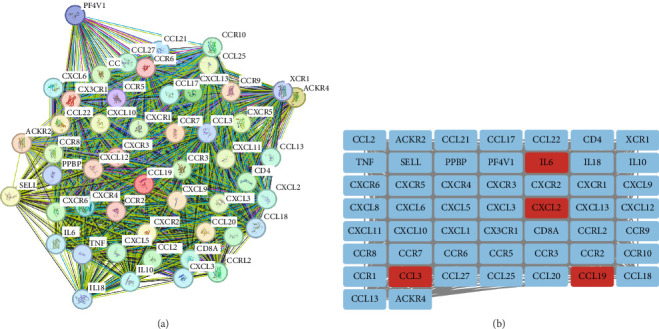
Interaction network of CCL19 and related genes in IgAN.

Using the GSE116626 dataset and applying the filtering criteria of |LogFC| ≥ 1.5 and *p*  < 0.05, a total of 486 DEGs associated with IgAN were identified. The intersection of these DEGs with the MR results yielded a single overlapping gene, CCL19. Statistical analysis of this gene was performed using GraphPad Prism, and the results from Figure 4 demonstrated a significant upregulation of CCL19 in renal tissues of IgAN patients. Furthermore, integrating the results from the PPI network with the DEG analysis revealed interactions between CCL19 and C─C motif chemokine ligand 3 (CCL3), C─X─C motif chemokine ligand 2 (CXCL2), and IL6 (Figure 5).

## 4. Discussion

The risk factors for IgAN are numerous, but its exact pathogenesis remains unclear. It remains unclear whether cytokines contribute to the pathogenesis of IgAN or if they are simply clinical indicators of the disease. MR is widely employed to explore causal relationships between diseases and risk factors. In this study, the causal link between circulating inflammatory cytokines and IgAN was examined using a bidirectional two-sample MR analysis. The analysis revealed that elevated levels of TGF-alpha, LIF, and CCL19 are positively associated with an increased risk of IgAN. However, when IgAN was treated as the exposure variable, no significant associations with inflammatory cytokines were identified. An intersection of the MR findings with DEGs from the GEO database demonstrated that the CCL19 gene is significantly upregulated in renal tissues from IgAN patients, further confirming the critical role of CCL19 in IgAN pathogenesis.

CCL19 is a chemokine integral to the maintenance of immune surveillance, homeostasis, and developmental processes. Our previous study in a murine model of renal injury has revealed that CCL19 is predominantly secreted by fibroblastic reticular cells (FRCs). FRCs, which serve as key stromal cells within tertiary lymphoid organs (TLOs) [22], play an essential role in orchestrating the structural and functional organization of these ectopic lymphoid structures. TLOs recapitulate the architecture of secondary lymphoid follicles and are frequently observed in chronically inflamed tissues. Local expression of lymphoid neogenesis factors, particularly CCL19, is pivotal for TLO development [23]. Approximately, one-third of IgAN patients exhibit renal TLOs [24], and elevated serum CCL19 levels in these individuals further implicate CCL19 in driving TLO formation and the associated chronic inflammatory milieu.

Traditionally, CCL19 has been attributed primarily to dendritic cells, which regulate T-cell activation, immune tolerance, and inflammatory responses [25]. Dendritic cell-derived CCL19, via its interaction with the CCR7 receptor, mediates the recruitment of various immune cells to the kidney [26]. In IgA nephropathy, the crosstalk between dendritic cells and T cells is critical for T-cell activation, with subsequent functional alterations underpinning the immunopathogenesis of the disease [27]. Moreover, CCL19 has been shown to enhance local immune responses by promoting dendritic cell maturation, thereby stimulating T-cell proliferation and upregulating costimulatory molecules and proinflammatory cytokines [28, 29]. In synergy with adhesion molecules such as Vascular Cell Adhesion Molecule-1 (VCAM-1) and Intercellular Adhesion Molecule-1 (ICAM-1), CCL19 facilitates immune cell adhesion and migration, prolonging the residency of both dendritic cells and T cells within inflamed renal tissue [30]. In addition, chemokines, such as CCL3 and CXCL2, recruit neutrophils and other inflammatory cells to sites of injury, further amplifying local immune activation [31], while elevated levels of IL-6 correlate with increased disease activity and progression in IgAN [4]. Collectively, these interactions underscore a complex regulatory network that modulates the renal immune microenvironment and may contribute to the progression of IgAN.

In addition, B cell activating factor (BAFF) plays a significant role in IgAN patients. Transgenic mice overexpressing BAFF exhibit an IgAN-like renal phenotype [32]. Anti-BAFF therapy markedly decreases the development of renal B cell zones and TLOs, along with the expression of factors that promote TLO formation and markers of B cell differentiation originating from renal B cells [33]. This suggests that TLO development and enhanced local adaptive immune responses are promoted by renal B cells that are dependent on BAFF. We infer that the high expression levels of CCL19 within and around TLOs facilitate the recruitment and co-localization of T and B cells, creating an optimal microenvironment for BAFF to effectively promote B cell activation and excessive glycosylated IgA1 (Gd-IgA1) production [34], thereby exacerbating the inflammatory response in IgAN patients. Given the study's findings that CCL19 is positively correlated with IgAN risk, it is hypothesized that CCL19 may contribute to the exacerbation of IgAN pathogenesis by promoting CCL19-dependent B cell activation and TLO formation.

TGF-alpha is extensively expressed in normal adult tissues [35] and belongs to the epidermal growth factor (EGF) family including the skin, kidneys, liver, breast, eosinophils, and macrophages. In rodent models of chronic kidney disease (CKD), TGF-alpha has been shown to promote renal parenchymal deterioration through epidermal growth factor receptor (EGFR) activation [36]. Studies have found the expression of Ang II type 2 receptor (AT2R) in renal tubules is increased in IgAN [37]. TGF-alpha is a key mediator of AngII-induced kidney injury, and basic research has demonstrated the importance of the TACE-TGF-alpha-EGFR pathway in AngII-induced kidney injury [38]. Clinical renal biopsies from IgAN patients have revealed that an increased risk of IgAN progression is associated with elevated intrarenal AngII expression [39]. It is hypothesized that TGF-alpha may play a role in the initiation and advancement of IgAN by affecting the renin–angiotensin–aldosterone system.

LIF, a member of the IL-6 family, is expressed in mucosal tissues and possesses immunomodulatory functions [40]. It is a non-invasive biomarker of CKD progression and a potential therapeutic target for renal tubulointerstitial fibrosis [41]. Flesh-eye hematuria in patients with IgAN was found to be strongly associated with upper respiratory tract infections, suggesting that there may be an important link between IgAN and mucosa-associated lymphoid tissue [42]. In IgAN, the patient's serum and glomerular immune deposits contain high levels of improperly Gd-IgA1 [43]. IgA1-secreting cells produce Gd-IgA1 molecules, with pathogenic aberrant O-glycosylation being closely linked to the pathological activity of IgAN and alterations in renal function [44]. Environmental factors, such as mucosal infections, may further exacerbate the iso-O-glycosylation of IgA1 in a cytokine-mediated manner. The role of LIF in the pathogenesis of IgAN is further supported by studies showing that LIF can increase Gd-IgA1 production in IgA1-producing cell lines of IgAN patients [45]. In addition, the region on chromosome 22q12, which includes several candidate genes, including the gene encoding LIF, has also been found to affect serum IgA levels [46]. In conclusion, LIF, as a member of the IL-6 family, affects IgA levels and the development of IgAN by influencing the aberrant O-glycosylation of IgA1 and by gene regulatory mechanisms.

No significant inflammatory cytokines were detected when reversing exposure and outcome and IgAN was considered as an exposure variable. Causality in biological systems is complex, and multifactorial and multi-pathway interactions may mask the detection of single reverse causality, and the presence of certain mediating variables or reverse pathways may not be adequately considered.

However, some limitations of this study must be discussed: Initially, a significance threshold of *p*=5 × 10^−6^ was applied to the exposed GWAS data. This adjustment was made because the number of genome-wide significant SNPs was minimal at the *p*  < 5 × 10^−8^ level, and thus it became challenging to perform further MR analyses. Second, in this study, the estimates obtained from MR-Egger, weighted median, simple mode, and weighted mode methods did not achieve statistical significance. Given the higher statistical power of the IVW method compared to other MR analyses, along with the consistency in the direction of the beta coefficients in this study, the findings can also be regarded as significant. The third problem is that the study's GWAS data were only drawn from European populations, which raises the possibility that people with different ancestries may not be able to exploit the study's findings. Finally, TGF-alpha and LIF were not shown to be positive in the GEO database. More studies are needed to confirm the results of this study, and these findings should be leveraged in clinical practice to inform the development of diagnostic and therapeutic strategies.

## 5. Conclusions

Our research indicates that three inflammatory factors, such as TGF-alpha, LIF, and CCL19, may be upstream in the pathogenic pathway of IgAN, and the key role of CCL19 in IgAN pathogenesis was further validated by the GEO database, but whether these cytokines can be used to predict or ameliorate the progression of IgAN needs to be further investigated.

## Figures and Tables

**Table 1:** Results of heterogeneity and sensitivity analysis of 91 inflammatory factors on the causal effect of IgAN

Exposure	Q_Inverse.variance.weighted	Q_df_Inverse.variance.weighted	Q_pval_Inverse.variance.weighted	egger_intercept	se	pval	outliers	global_test_p
Transforming growth factor-alpha levels	4.196010855	8	0.839019685	0.026237448	0.057334377	0.661089706	—	0.847
Leukemia inhibitory factor levels	5.050890046	6	0.537302873	0.005034178	0.070521182	0.945858465	—	0.627
C–C motif chemokine 19 levels	11.81430471	12	0.460706076	0.06855105	0.044086358	0.1482467	—	0.532
C–C motif chemokine 20 levels	20.09792893	15	0.16820812	0.096141524	0.059654479	0.129348405	NA	0.005
Programmed cell death 1 ligand 1 levels	14.27916872	11	0.217929037	−0.021502125	0.057396208	0.71576063	—	0.264
Monocyte chemoattractant protein-3 levels	13.24214544	15	0.58360083	−0.013176375	0.046134393	0.779360141	—	0.622
Interleukin-13 levels	4.795079875	9	0.851793242	−0.033590022	0.054509053	0.554868632	—	0.87

## Data Availability

The data supporting this research are sourced from the FINNGEN database and the GEO database. The authors sincerely thank the relevant researchers for sharing the GWAS summary statistics included in this study. All the aforementioned data are publicly available.

## Supporting Information

Supporting Information Supporting Information Table S1: Information on 91 inflammatory factors. Figure S1: Programmed cell death 1 ligand 1 levels (GCST90274832) and Interleukin-13 levels (GCST90274799), Monocyte chemoattractant protein-3 levels (GCST90274823) with a leave-one-out plot of the causal relationship between IgAN.
