# Supplementary material for: Exploring the Relationship Between 91 Inflammatory Cytokines and IgA Nephropathy Using a Two-Sample Mendelian Randomization Study and the Gene Expression Omnibus Database
Source: Mediators Inflamm. 2025 Apr 26;2025:5142090. doi: 10.1155/mi/5142090 (PMC12049251; doi:10.1155/mi/5142090)

Supplementary Table 1： Information on 91 inflammatory factors

| Reported Trait | symbol | Efo Traits | samples | id | F |
| --- | --- | --- | --- | --- | --- |
| Eukaryotic translation initiation factor 4E-binding protein 1 levels | 4EBP1 | level of eukaryotic translation initiation factor 4E-binding protein 1 in blood plasma | 14736 | GCST90274758 | 35.66872804 |
| Adenosine Deaminase levels | ADA | adenosine deaminase measurement | 14736 | GCST90274759 | 159.476816 |
| Artemin levels | ARTN | artemin measurement | 11778 | GCST90274760 | 22.32078599 |
| Axin-1 levels | AXIN1 | axin-1 measurement | 11793 | GCST90274761 | 23.37995894 |
| beta-nerve growth factor levels | Beta-NGF | beta-nerve growth factor measurement | 14743 | GCST90274762 | 24.85797558 |
| Caspase 8 levels | CASP-8 | caspase-8 measurement | 14744 | GCST90274763 | 22.9504033 |
| Eotaxin levels | CCL11 | eotaxin measurement | 14734 | GCST90274764 | 43.60485085 |
| C-C motif chemokine 19 levels | CCL19 | C-C motif chemokine 19 measurement | 14736 | GCST90274765 | 37.4638491 |
| C-C motif chemokine 20 levels | CCL20 | C-C motif chemokine 20 measurement | 14736 | GCST90274766 | 27.35581996 |
| C-C motif chemokine 23 levels | CCL23 | C-C motif chemokine 23 measurement | 14736 | GCST90274767 | 82.99843649 |
| C-C motif chemokine 25 levels | CCL25 | C-C motif chemokine 25 measurement | 14736 | GCST90274768 | 83.87775627 |
| C-C motif chemokine 28 levels | CCL28 | C-C motif chemokine 28 measurement | 14734 | GCST90274769 | 25.68983606 |
| C-C motif chemokine 4 levels | CCL4 | C-C motif chemokine 4-like measurement | 14744 | GCST90274770 | 124.1611857 |
| Natural killer cell receptor 2B4 levels | CD244 | natural killer cell receptor 2B4 measurement | 14735 | GCST90274771 | 43.33648511 |
| CD40L receptor levels | CD40 | CD40 measurement | 14736 | GCST90274772 | 102.2072383 |
| T-cell surface glycoprotein CD5 levels | CD5 | t-cell surface glycoprotein CD5 measurement | 14735 | GCST90274773 | 31.56769184 |
| T-cell surface glycoprotein CD6 isoform levels | CD6 | level of T-cell differentiation antigen CD6 in blood plasma | 14735 | GCST90274774 | 158.5807735 |
| CUB domain-containing protein 1 levels | CDCP1 | CUB domain-containing protein 1 measurement | 14734 | GCST90274775 | 36.57137043 |
| Macrophage colony-stimulating factor 1 levels | CSF-1 | macrophage colony-stimulating factor 1 measurement | 14734 | GCST90274776 | 36.60996827 |
| Cystatin D levels | CST5 | cystatin-D measurement | 14736 | GCST90274777 | 65.17419122 |
| Fractalkine levels | CX3CL1 | fractalkine measurement | 14743 | GCST90274778 | 26.51527362 |
| C-X-C motif chemokine 1 levels | CXCL1 | CXCL1 measurement | 14736 | GCST90274779 | 96.36804669 |
| C-X-C motif chemokine 10 levels | CXCL10 | C-X-C motif chemokine 10 measurement | 14744 | GCST90274780 | 36.69834741 |
| C-X-C motif chemokine 11 levels | CXCL11 | C-X-C motif chemokine 11 measurement | 14736 | GCST90274781 | 37.80596804 |
| C-X-C motif chemokine 5 levels | CXCL5 | C-X-C motif chemokine 5 measurement | 14736 | GCST90274782 | 97.54824563 |
| C-X-C motif chemokine 6 levels | CXCL6 | C-X-C motif chemokine 6 measurement | 14744 | GCST90274783 | 159.0403205 |
| C-X-C motif chemokine 9 levels | CXCL9 | C-X-C motif chemokine 9 measurement | 14735 | GCST90274784 | 32.1166784 |
| Delta and Notch-like epidermal growth factor-related receptor levels | DNER | delta and Notch-like epidermal growth factor-related receptor measurement | 14735 | GCST90274785 | 33.21001619 |
| Protein S100-A12 levels | EN-RAGE | protein S100-A12 measurement | 14743 | GCST90274786 | 34.40657087 |
| Fibroblast growth factor 19 levels | FGF-19 | fibroblast growth factor 19 measurement | 14744 | GCST90274787 | 34.34279492 |
| Fibroblast growth factor 21 levels | FGF-21 | fibroblast growth factor 21 measurement | 14743 | GCST90274788 | 45.17735196 |
| Fibroblast growth factor 23 levels | FGF-23 | fibroblast growth factor 23 measurement | 14735 | GCST90274789 | 23.78237308 |
| Fibroblast growth factor 5 levels | FGF-5 | fibroblast growth factor 5 measurement | 11789 | GCST90274790 | 79.41896714 |
| Fms-related tyrosine kinase 3 ligand levels | FIt3L | obsolete_Fms-related tyrosine kinase 3 ligand measurement | 14734 | GCST90274791 | 47.12020646 |
| Glial cell line-derived neurotrophic factor levels | hGDNF | glial cell line-derived neurotrophic factor measurement | 14736 | GCST90274792 | 63.71400959 |
| Hepatocyte growth factor levels | HGF | hepatocyte growth factor measurement | 14734 | GCST90274793 | 30.34387766 |
| Interferon gamma levels | IFN-gamma | interferon gamma measurement | 11793 | GCST90274794 | 24.30091376 |
| Interleukin-10 levels | IL-10 | interleukin-10 measurement | 14744 | GCST90274795 | 30.97111144 |
| Interleukin-10 receptor subunit alpha levels | IL-10RA | interleukin-10 receptor subunit alpha measurement | 11793 | GCST90274796 | 22.65125267 |
| Interleukin-10 receptor subunit beta levels | IL10RB | interleukin-10 receptor subunit beta measurement | 14734 | GCST90274797 | 101.2570285 |
| Interleukin-12 subunit beta levels | IL-12B | obsolete_interleukin-12 subunit B measurement | 14735 | GCST90274798 | 111.0119494 |
| Interleukin-13 levels | IL-13 | interleukin-13 measurement | 11792 | GCST90274799 | 23.98525414 |
| Interleukin-15 receptor subunit alpha levels | IL-15RA | interleukin-15 receptor subunit alpha measurement | 11792 | GCST90274800 | 135.8444982 |
| Interleukin-17A levels | IL-17A | interleukin-17A measurement | 11784 | GCST90274801 | 22.01782653 |
| Interleukin-17C levels | IL-17C | interleukin-17C measurement | 11793 | GCST90274802 | 25.08646675 |
| Interleukin-18 levels | IL-18 | interleukin 18 measurement | 14744 | GCST90274803 | 53.8557065 |
| interleukin-18 receptor 1 levels | IL-18R1 | interleukin-18 receptor 1 measurement | 14743 | GCST90274804 | 70.88357807 |
| Interleukin-1-alpha levels | IL-1 alpha | obsolete_interleukin-1 alpha measurement | 11788 | GCST90274805 | 35.87614396 |
| Interleukin-2 levels | IL-2 | interleukin-2 measurement | 11789 | GCST90274806 | 22.1334153 |
| Interleukin-20 levels | IL-20 | interleukin-20 measurement | 11784 | GCST90274807 | 23.0637594 |
| Interleukin-20 receptor subunit alpha levels | IL-20RA | interleukin-20 receptor subunit alpha measurement | 11792 | GCST90274808 | 23.7316684 |
| Interleukin-22 receptor subunit alpha-1 levels | IL-22RA1 | interleukin-22 receptor subunit alpha-1 measurement | 11793 | GCST90274809 | 22.04853753 |
| Interleukin-24 levels | IL-24 | interleukin-24 measurement | 11785 | GCST90274810 | 22.69178808 |
| Interleukin-2 receptor subunit beta levels | IL-2RB | interleukin-2 receptor subunit beta measurement | 11792 | GCST90274811 | 22.516472 |
| Interleukin-33 levels | IL-33 | level of interleukin-33 in blood plasma | 11793 | GCST90274812 | 22.34191228 |
| Interleukin-4 levels | IL-4 | interleukin-4 measurement | 11793 | GCST90274813 | 23.16616431 |
| Interleukin-5 levels | IL-5 | interleukin-5 measurement | 11792 | GCST90274814 | 22.9750457 |
| Interleukin-6 levels | IL-6 | interleukin-6 measurement | 14743 | GCST90274815 | 45.04059641 |
| Interleukin-7 levels | IL-7 | interleukin-7 measurement | 14736 | GCST90274816 | 23.04982202 |
| Interleukin-8 levels | IL-8 | interleukin-8 measurement | 14744 | GCST90274817 | 26.26617789 |
| Latency-associated peptide transforming growth factor beta 1 levels | LAP TGF-beta-1 | transforming growth factor beta-1 measurement | 14736 | GCST90274818 | 33.72532649 |
| Leukemia inhibitory factor levels | LIF | leukemia inhibitory factor measurement | 11793 | GCST90274819 | 22.51401813 |
| Leukemia inhibitory factor receptor levels | LIF-R | leukemia inhibitory factor receptor measurement | 11784 | GCST90274820 | 48.57937334 |
| Monocyte chemoattractant protein-1 levels | CCL2 | CCL2 measurement | 14733 | GCST90274821 | 45.40892506 |
| Monocyte chemoattractant protein 2 levels | CCL8 | monocyte chemotactic protein-2 measurement | 14736 | GCST90274822 | 127.6703013 |
| Monocyte chemoattractant protein-3 levels | CCL7 | monocyte chemotactic protein 3 measurement | 11783 | GCST90274823 | 35.3898353 |
| Monocyte chemoattractant protein-4 levels | CCL13 | monocyte chemotactic protein-4 measurement | 14736 | GCST90274824 | 67.27205889 |
| Macrophage inflammatory protein 1a levels | MIP-1 alpha | macrophage inflammatory protein 1a measurement | 14743 | GCST90274825 | 101.586342 |
| Matrix metalloproteinase-1 levels | MMP-1 | matrix metalloproteinase 1 measurement | 14744 | GCST90274826 | 42.65007 |
| Matrix metalloproteinase-10 levels | MMP-10 | matrix metalloproteinase 10 measurement | 14744 | GCST90274827 | 102.5365241 |
| Neurturin levels | NRTN | level of neurturin in blood plasma | 11791 | GCST90274828 | 22.53327666 |
| Neurotrophin-3 levels | NT-3 | neurotrophin-3 measurement | 14744 | GCST90274829 | 22.3011359 |
| Osteoprotegerin levels | OPG | osteoprotegerin measurement | 14733 | GCST90274830 | 47.20838022 |
| Oncostatin-M levels | OSM | oncostatin-M measurement | 14736 | GCST90274831 | 32.99290835 |
| Programmed cell death 1 ligand 1 levels | PD-L1 | programmed cell death 1 ligand 1 measurement | 14736 | GCST90274832 | 31.6151863 |
| Stem cell factor levels | SCF | stem Cell Factor measurement | 14736 | GCST90274833 | 43.59379735 |
| SIR2-like protein 2 levels | SIRT2 | SIR2-like protein 2 measurement | 14736 | GCST90274834 | 31.48551482 |
| Signaling lymphocytic activation molecule levels | SLAMF1 | signaling lymphocytic activation molecule measurement | 14734 | GCST90274835 | 35.43930367 |
| Sulfotransferase 1A1 levels | ST1A1 | sulfotrasferase 1A1 measurement | 11793 | GCST90274836 | 38.64342374 |
| STAM binding protein levels | STAMPB | STAM binding protein measurement | 14736 | GCST90274837 | 23.33034943 |
| Transforming growth factor-alpha levels | TGF-alpha | transforming growth factor-alpha measurement | 14733 | GCST90274838 | 26.10130079 |
| Tumor necrosis factor levels | TWEAK | tumor necrosis factor measurement | 11785 | GCST90274839 | 23.2150163 |
| TNF-beta levels | TNFB | lymphotoxin-alpha measurement | 11792 | GCST90274840 | 84.07055969 |
| Tumor necrosis factor receptor superfamily member 9 levels | TNFRSF9 | tumor necrosis factor receptor superfamily member 9 measurement | 11784 | GCST90274841 | 27.3869411 |
| Tumor necrosis factor ligand superfamily member 14 levels | TNFSF14 | tumor necrosis factor ligand superfamily member 14 measurement | 11793 | GCST90274842 | 38.95353425 |
| TNF-related apoptosis-inducing ligand levels | TRAIL | TNF-related apoptosis-inducing ligand measurement | 14735 | GCST90274843 | 74.05407697 |
| TNF-related activation-induced cytokine levels | TRANCE | TNF-related activation-induced cytokine measurement | 14736 | GCST90274844 | 47.48467187 |
| Thymic stromal lymphopoietin levels | TSLP | thymic stromal lymphopoietin measurement | 11793 | GCST90274845 | 23.08787409 |
| Tumor necrosis factor ligand superfamily member 12 levels | TNF | tumor necrosis factor ligand superfamily member 12 measurement | 14736 | GCST90274846 | 38.98442595 |
| Urokinase-type plasminogen activator levels | uPA | urokinase-type plasminogen activator measurement | 14734 | GCST90274847 | 42.8928981 |
| Vascular endothelial growth factor A levels | VEGF_A | vascular endothelial growth factor A measurement | 14744 | GCST90274848 | 79.75010954 |

Figure S1：Programmed cell death 1 ligand 1 levels (GCST90274832) and Interleukin-13 levels (GCST90274799), Monocyte chemoattractant protein-3 levels ( GCST90274823) with a leave-one-out plot of the causal relationship between IgAN.


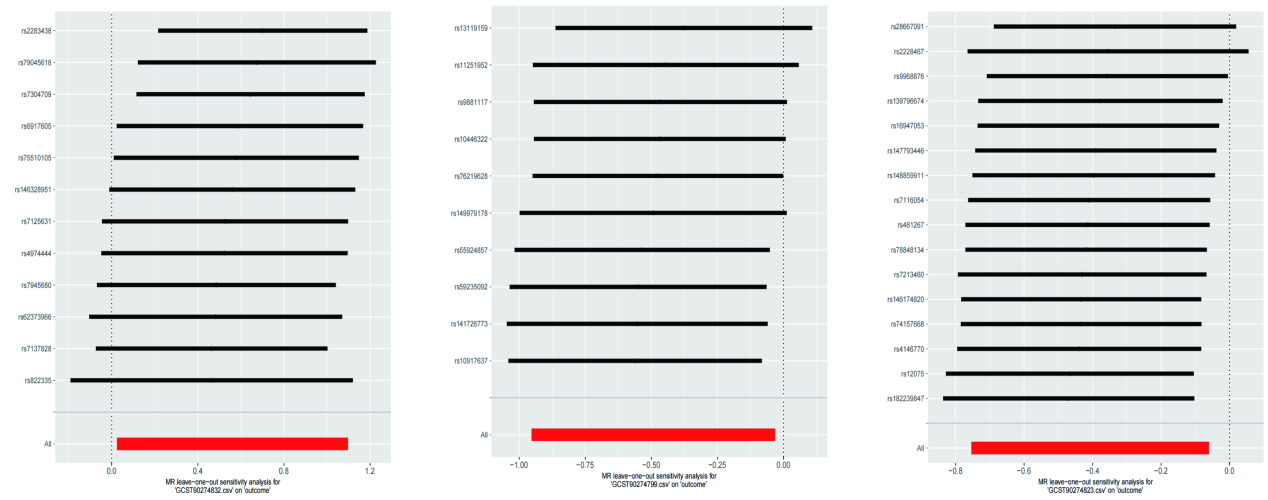

Supplement: Supporting Information — Table S1: Information on 91 inflammatory factors. Figure S1: Programmed cell death 1 ligand 1 levels (GCST90274832) and Interleukin-13 levels (GCST90274799), Monocyte chemoattractant protein-3 levels (GCST90274823) with a leave-one-out plot of the causal relationship between IgAN. [file 5142090.f1.docx]
